# The First Report on Liver Resection Using the Novel Japanese hinotori™ Surgical Robot System: First Case Series Report of 10 Cases

**DOI:** 10.3390/jcm13247819

**Published:** 2024-12-21

**Authors:** Kenichi Nakamura, Tetsuya Koide, Takahiko Higashiguchi, Kazuhiro Matsuo, Tomoyoshi Endo, Kenji Kikuchi, Koji Morohara, Hidetoshi Katsuno, Ichiro Uyama, Koichi Suda, Zenichi Morise

**Affiliations:** 1 Department of Surgery, Fujita Health University Okazaki Medical Center, 1 Gotanda, Harisaki-cho, Okazaki 444-0827, Aichi, Japan; naka-ken@fujita-hu.ac.jp (K.N.); tetsuya.koide@fujita-hu.ac.jp (T.K.); thigashi@fujita-hu.ac.jp (T.H.); kzhrmto@fujita-hu.ac.jp (K.M.); tomtom23@fujita-hu.ac.jp (T.E.); kiku0414@fujita-hu.ac.jp (K.K.); koji.morohara@fujita-hu.ac.jp (K.M.); katsuno@fujita-hu.ac.jp (H.K.); iuyama@fujita-hu.ac.jp (I.U.); 2 Department of Surgery, Fujita Health University, 1-98 Dengakugakubo, Kutsukake, Toyoake 470-1192, Aichi, Japan; ko-suda@fujita-hu.ac.jp; 3 Department of Advanced Robotic and Endoscopic Surgery, Fujita Health University, 1-98 Dengakugakubo, Kutsukake, Toyoake 470-1192, Aichi, Japan

**Keywords:** liver resection, robot surgery, laparoscopic liver resection, robotic liver resection

## Abstract

**Background:** In Japan, the hinotori™ surgical robot system (Medicaroid Corporation, Kobe, Japan) was approved for gastrointestinal surgeries in October 2022. This report details our initial experience performing liver resection using the hinotori™ system. **Methods:** Ten patients, who were assessed as cases that would benefit from the robot-assisted procedure, underwent liver resections using the hinotori™ system at Fujita Health University, Okazaki Medical Center, between August 2023 and October 2024. The backgrounds (patient, tumor, and liver function conditions, along with types of liver resections and previous surgical procedures) and short-term outcomes (operation time, blood loss, postoperative complications, open conversion, length of hospital stay, and mortality) of the cases were evaluated. **Results:** Eight cases of partial liver resection, one extended left medial sectionectomy, and one left hemi-hepatectomy were performed. Six cases of hepatocellular carcinomas, three cases of liver metastases, and one case of hepatolithiasis were included. There were seven male and three female patients with a median age of 70 years. Three physical status class III and seven class II patients were included. The median body mass index was 24. Five patients had previous upper abdominal surgical histories and five patients had liver cirrhosis. The median operation time was 419.5 min, and the median intraoperative blood loss was 276 mL. An open conversion in one hepatocellular carcinoma case was carried out due to bleeding from collateral vessels in the round ligament. The median length of hospital stay was 7.5 days. A grade IIIa complication (delayed bile leakage) was developed in one case. All patients with tumors underwent R0 resection. There were no cases of mortality. **Conclusions:** Liver resection using the hinotori™ system was feasibly performed. This study reports the first global use of the hinotori™ system for liver resection.

## 1. Introduction

The hinotori™ surgical robot system (hinotori) is a Japanese surgical-assistance robot developed by the Medicaroid Corporation (Kobe, Japan), a joint venture between the Sysmex Corporation (Kobe, Japan) and Kawasaki Heavy Industries, Ltd. (Kobe, Japan).

It received initial regulatory approval from the Pharmaceuticals and Medical Devices Agency of Japan in 2020 for clinical applications in urology. Subsequently, approval for its use in gastrointestinal surgery was obtained in October 2022. In recent years, there have been reports on the application of the hinotori system in gastrointestinal surgery, specifically gastric, colorectal, and pancreatic surgeries [1,2,3,4,5,6,7,8], and overseas expansion has recently begun in Singapore.

The hinotori liver resection program was started by surgeon Z.M., who has experience performing more than 300 laparoscopic liver resections, although he had no experience with robot-assisted surgeries at all. The initial cases selected were assessed as being moderately complicated procedures with possible benefits to be gained from robot assistance. This study and review are the world’s first report on hinotori liver surgery and describe our initial experiences.

## 2. Materials and Methods—Case Series Examination

A retrospective observational examination was conducted to evaluate the safety and feasibility of our liver resection program using hinotori. The first 10 consecutive patients, who were assessed preoperatively as cases with moderate complexity that would possibly benefit from a robot-assisted procedure, underwent liver resections using hinotori at Fujita Health University, Okazaki Medical Center, between August 2023 and October 2024. The backgrounds (patient, tumor, and liver function conditions, along with types of liver resections and previous surgical procedures) and short-term outcomes of the cases were evaluated.

The liver surgeon Z.M., an experienced surgeon who had performed more than 300 cases of laparoscopic liver resection but who had no experience with robot-assisted surgeries at all, was the main surgeon for these cases. In the first case of liver surgery, he was the main assistant and instructor for a surgeon experienced in robot-assisted procedures (I.U.). In the next three cases, the surgeon with experience in robot-assisted operations (I.U.) was the advisor for the liver surgeon (Z.M.) who acted as the robot operator. The liver surgeon performed the surgeries independently in all remaining cases. For all cases, supporting staff, such as bedside surgeons, medical engineers, and nurses, were highly experienced in robot-assisted surgeries using both hinotori and the da Vinci^TM^ Surgical System (da Vinci, Intuitive Surgical Inc., Sunnyvale, CA, USA).

We collected data from the prospective database and electronic medical records maintained at our institution; these included clinical data on patient demographics, preoperative assessments, surgical outcomes, and pathological results. Surgical outcomes, such as operation time, blood loss, and postoperative complications in each case, were also prospectively monitored during the study in order to prevent any adverse effects. All patients were treated according to the Declaration of Helsinki, and this study was conducted after obtaining informed consent from each patient for the surgery and the study. The study was also approved by the Institutional Review Board (HM24-029).

### 2.1. Characteristics of the Patients and Short-Term Outcomes

The characteristics (age; gender; body mass index; physical status classification according to the American Society of Anesthesiologists (ASA-PS); diagnoses requiring liver resection; the presence of liver fibrosis/cirrhosis and portal hypertension; previous surgical history; number and size of tumors; location of tumors; and types of liver resections, including the need for major vessel dissections) and short-term outcomes (operation time, blood loss, open conversion, postoperative complications, length of hospital stay, and mortality) of the first 10 consecutive patients were evaluated.

### 2.2. hinotori™ Surgical Robot System

Hinotori is similar in design to da Vinci, which has four robotic arms and similar types of forceps. However, hinotori includes robotic arms with eight axes of motion, one more than da Vinci, allowing for more flexibility of arm movement and minimizing the risk of interference between the arms [9,10,11].

**Figure 1:**
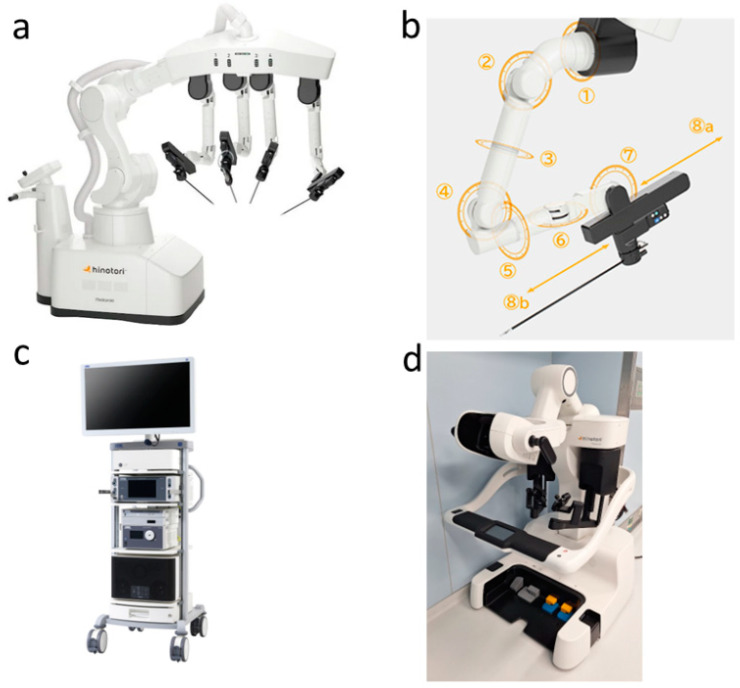
The hinotori™ Surgical Robot System. (**a**) The operation unit with four robotic arms; the Hinotori™ Surgical Robot System has four arms, similar to those of the da Vinci™ Surgical System. However, the manipulating arms do not require docking with the ports. (**b**) Robotic arms with eight axes of motion. These arms have one more axis than the arms of the da Vinci™ Surgical System, allowing flexibility of arm movement and minimizing the risk of interference between the arms. (**c**) Monitor cart and (**d**) surgeon cockpit. The surgeon’s cockpit features a flexible 3D viewer that helps reduce neck and shoulder fatigue. The operating procedure is similar to that of the da Vinci™ Surgical System.

**Figure 2:**
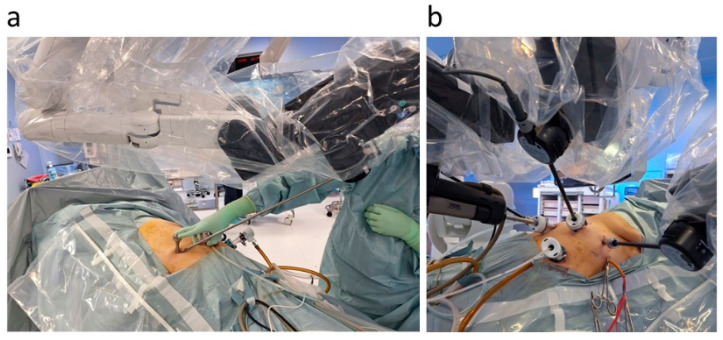
The docking-free design of hinotori. (**a**) Pivoting using a pivoter. The pivot point (the center of the movement on the abdominal wall) of the instruments is controlled via software. Therefore, no docking of the port and arm is required. This has the potential to reduce damage to the abdominal wall caused by port traction. (**b**) A large working space around the port. There is no docking of the port, and the arm provides more space around the port, making external manipulation easier.

**Figure 3:**
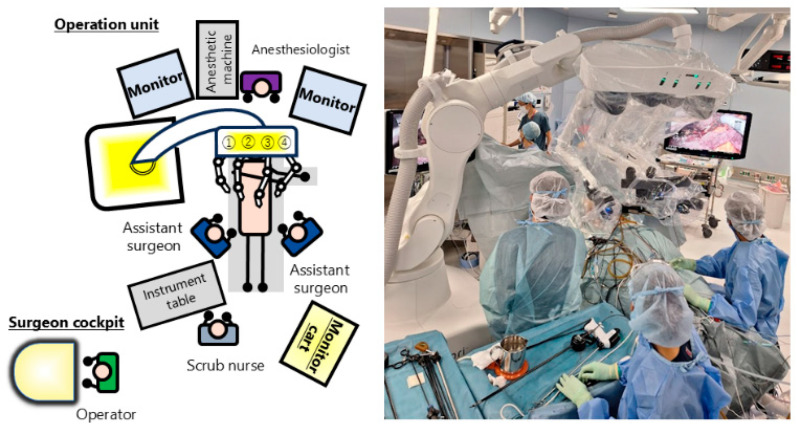
Operating theater configuration. There are two assistant surgeons beside the patient who perform the extracorporeal intermittent Pringle maneuver, change the robot’s instruments, etc. In addition, the hinotori™ Surgical Robot System does not have a vessel sealing system and suction–irrigation device; thus, the assistant surgeons use laparoscopic devices to assist with the surgery.

Furthermore, hinotori features a docking-free design, with the pivot point (the center of the movement on the port site in the abdominal wall) of the instruments controlled via software (Figure 1, Figure 2 and Figure 3) [7,9]. The da Vinci arm grasps the trocar, whereas hinotori does not. This allows for a large area around the port and has the potential to reduce damage to the abdominal wall caused by port traction [10,11]. On the other hand, unlike da Vinci, original sealing devices, staplers, suction/irrigation devices, and dual consoles have not yet been developed for hinotori [12].

### 2.3. Operative Procedures

The patient was placed in the supine or right lateral decubitus position. A 12 mm port with a balloon was placed in the umbilicus, and the abdominal cavity was observed under a pneumo-peritoneum pressure of 10 mm Hg, followed by the insertion of five ports in the upper abdomen. The placement of the ports was decided depending on the tumor location, resection style, and adhesion from previous surgery in each case. Our usual port placements during laparoscopic liver resection were described previously [13]. However, since robot ports should be at least 6.5 cm apart from each other and the distance between the ports and the target lesion should also be longer than the laparoscopic setting, the ports in this series were generally placed at more caudal sites, and they were placed further apart from each other. Although hinotori already has a re-usable 8 mm metallic port, conventional ports for laparoscopic surgery can also be used. For the first port and the camera port, we used a conventional disposable 12 mm port with a balloon. A tube and a tape were inserted in the left subcostal area for the Pringle maneuver, if applicable. After all the ports were set, the patient was placed in a 12-degree head-up position, and hinotori was used for the surgery.

A pair of fenestrated bipolar forceps (Medicaroid Inc., Kobe, Japan) were used in the left hand of the main surgeon and inserted at the right-most 8 mm port. A laparoscope was inserted at the second-right 12 mm port with a balloon. Monopolar curved scissors (Medicaroid Inc., Kobe Japan), Maryland bipolar forceps (Medicaroid Inc., Kobe, Japan), Clip Appliers (Medicaroid Inc., Kobe, Japan), or Wide Needle Holders (Medicaroid Inc., Kobe, Japan) for the main right hand of the surgeon were inserted at the left-side 8 mm port next to the camera port. A universal grasper (Medicaroid Inc., Kobe, Japan) for the right hand of the surgeon was inserted at the left-most 8 mm port as a traction sub-arm. Because a vessel sealing system and suction–irrigation device were not available for hinotori, the assistant surgeons used Sonicision™ (Medtronic Inc., Minneapolis, MN, USA) for dividing small vessels, and BiClamp^®^ (Erbe Elektromedizin, Tübingen, Germany) and an IO advance electrode (AMCO, Nagoya, Japan) were used for hemostasis. A laparoscopic suction–irrigation device (premium angle suction–irrigator, Heiwa Medical Instrument Co., Ltd., Hofu, Japan) was also used by the assistant surgeons. The devices used by the assistant surgeons were installed in the port in the umbilicus or in the other port placed between the main surgeon’s ports.

Liver parenchymal transection was mainly performed by crushing, sweeping, and dividing the liver tissue with the closed curved scissors or the Maryland bipolar forceps installed in the main right hand of the robot. The extent of liver resection was determined according to the Makuuchi criteria [14]. Multi-detector row computed tomography images of the liver, including the tumor, were reconstructed via 3D preoperative simulations using Revoras^TM^ software (1st version, Ziosoft Inc., Minato, Japan); liver resection was performed on the basis of these images and confirmed via intraoperative ultrasound, with the Glissonian sheath and hepatic veins used as landmarks. The extracorporeal intermittent Pringle maneuver was applied, with 15 min clamping time interrupted by 5 min of reperfusion for each patient, except in cases 6 and 8 where hepato-duodenal ligament encirclement could not be realized due to the thick adhesion from open pancreatoduodenectomy and 3 previous surgeries.

## 3. Results

The characteristics of the 10 cases are presented in Table 1.

The cases were selected as cases with possible advantages to the use of robots due to the dexterity of the bendable hands, eliminating physiological tremors and providing an extra-magnified stable three-dimensional view. Each case was a planned surgery, and they comprised the following reasons for robot-assisted surgery:

1. The need for major vessel dissection in deep areas of the liver (cases with deep tumors sticking to major vessels and poor liver function resulting in intolerance relative to larger resection, and a case with a critical need for the resection of the bile duct stenosis area, which was suspected of malignancy);
2. The target lesion was difficult to access when using the rigid straight devices of conventional laparoscopic surgery (cases of a tumor in dorsal segment 7–8 and cases with tumors behind thick adhesions).

Eight cases of partial liver resection, one extended left medial sectionectomy, and one left hemi-hepatectomy were performed. There were seven males and three females with a median age of 70 (60–77; range). Three ASA-PS class III patients and seven class II patients were included. The median body mass index was 24 (15.2–31.2).

Five patients had previous upper abdominal surgical histories. Among them, there was one patient with a history of two liver resections and a right-sided colectomy with postoperative major anastomosis leakage. One case had a previous history of pancreatic cancer liver metastasis, and the patient underwent segment 3–4 partial resection behind the jejunum anastomoses after open pancreatoduodenectomy.

The procedure was applied to six hepatocellular carcinomas arising from liver cirrhosis/fibrosis backgrounds and three cases also exhibited portal hypertension, as indicated by esophageal varix formations or decreased platelet counts at less than 100,000/microliter of blood.

The short-term outcomes are shown in Table 2.

The median operation time was 419.5 minutes (260–743; range). The median intraoperative estimated blood loss was 276 mL (26–4081). The longest operation time (743 min) occurred in one HCC patient with thick adhesion who underwent partial resection of the liver segment 7-8, and the dorsal section underwent a third liver resection after a right colectomy with postoperative major anastomosis leakage. An open conversion in an HCC case (partial resection of dorsal segment 7-8) was experienced due to bleeding from the massive collateral vessels in the round ligament outside of the surgical area. This case was also the case with the largest volume of bleeding. The median postoperative length of hospital stay was 7.5 (5–23) days. In the case of extended left medial sectionectomy, a major postoperative complication (delayed bile leakage, IIIa in the Clavien–Dindo classification) was developed, and re-admission occurred. All patients with tumors received R0 resection. There was no mortality.

## 4. Discussion

This is the world’s first report of liver resections using a novel Japanese surgical robot system, hinotori, and this report describes 10 initial consecutive robotic liver resections. hinotori liver resection was shown to be feasible. All cases—assessed and selected preoperatively as cases that could most benefit from robot assistance—comprised moderately complicated procedures. They were safely completed by a liver surgeon (Z.M.) who had no experience with robot-assisted surgery, although he has carried out more than 300 cases of conventional laparoscopic liver resections. The supporting staff were experienced in robot-assisted surgeries. In four initial cases, a proctoring surgeon (I.U., who had experience in both robotic and liver surgery) supported the operations.

In our previous report [15] of 231 conventional laparoscopic liver resections, the operation time and intraoperative blood loss for all cases were 323 min and 108 mL (median). However, 133 of the 231 cases comprised simple partial resection and left lateral sectionectomy, and the cases selected for robotic procedures in the present report were more complicated cases. The operation time (420 min) and blood loss (276 mL) of the cases in the present report are similar to those (414 min and 255 mL) of cases excluding simple partial resection and left lateral sectionectomy. The data are also comparable to outcomes from previous reports for robotic procedures using da Vinci [16,17,18,19,20,21,22,23,24,25,26,27,28,29,30,31], although the operation time was relatively longer (Table 3 and Supplementary Table S1, including 16 reports for various cases of liver resection comparing robotic vs. laparoscopic/open surgery between 2019 and 2024 retrieved from Pubmed, Embase, and Cochrane Library, excluding systematic reviews and meta-analyses). The short-term operation time and blood loss outcomes could improve since the learning curve of the main surgeon (Z.M.) for robotic procedures might have had some impact on the results.

One case (10%) of open conversion was observed. The rate is similar to those of the right hemi-hepatectomy (8.3%), anterior sectionectomy (12.5%), and segment 7/8 segmentectomy (11.1%) cases in our previous report of conventional laparoscopic procedures [15]. Moreover, it was comparable to the outcomes from previous reports for robotic procedures using da Vinci, in which various cases of liver resection were included (Table 3 and Supplementary Table S1 [16,17,18,19,20,21,22,23,24,25,26,27,28,29,30,31]). Bleeding during the partial resection of dorsal segment 7–8 was caused by an injury to the massive collateral vessels in the round ligament, which was outside of the surgical view for dorsal segment 7–8. The ligament had been transected and pulled downward to the left in order to move the liver and gain enough surgical space in the right subphrenic space. Since the bleeding point could not be identified, the open conversion of the operation was selected. The rigid hinotori scope needed to be placed more cranially for the resection of dorsal segment 7–8, and this resulted in the visual loss of the bleeding area. In the da Vinci system, the camera port and the scope can be lifted up to a higher position by pulling up the abdominal wall; deeper views can be obtained via this method. This is not possible on the hinotori system due to the docking-free “PIVOT” design in which the port point is fixed and cannot be lifted up. Although the substantially magnified and stable view is one of the advantages of robot-assisted procedures, the bird’s-eye view of the entire surgical area is often traded off by the need to be cautious.

One postoperative bile leakage Clavien–Dindo (CD) grade IIIa case was observed. The rate (10%) of morbidity CD III (and also II) or more was comparable to the 7% rate for all cases in our previous report of conventional laparoscopic liver resection [15], and it was also within the range of the outcomes from previous reports for robotic procedures using da Vinci (Table 3, Supplementary Table S1 [16,17,18,19,20,21,22,23,24,25,26,27,28,29,30,31]). In this case, extended left medial sectionectomy was performed for the two metastatic tumors close to the umbilical portion of the Glissonian pedicle. Burned injury on the bile duct during the dissection of the umbilical Glissonian pedicle might have occurred via cautery, with original sealing devices not yet developed for hinotori [12]; ultrasonic surgical aspirators (CUSA)/water jets are also not equipped on robotic systems.

The length of postoperative hospital stay was shorter than in our previous report of conventional laparoscopic liver resection (14 days for all cases and 12 days for simple cases). A reduction in wound pain compared to da Vinci was reported during the hinotori radical prostatectomy as a result of less damage inflicted on the abdominal wall by the “PIVOT” mechanism, where the robotic arms move around the center point set on the port site [10]. Reduced wound pain may lead to earlier recovery and shorter hospital stays [32]. However, the hospital stay length of our conventional laparoscopic patients was relatively longer [15], and data were collected from patients over a long period. Since our hospitalization policy has changed gradually, with a tendency to shorten hospital stays, this point should be further examined prospectively.

The R status for tumors was well secured, and there was no mortality. Although long-term outcomes should be examined, hinotori liver resections in the present setting (for moderately complicated cases carried out by a surgeon inexperienced in robot-assisted surgery) were feasible.

There are an increasing number of reports of robot-assisted liver resection using da Vinci [33,34,35]. While da Vinci has long been highly competitive and the leading option for robotic surgery, several new platforms are now entering the field, and the Japanese hinotori robot is one of them. The studies comparing robot-assisted (da Vinci) resection to conventional laparoscopic liver resection generally exhibited possible advantages in short-term outcomes, such as less blood loss and shorter hospital stays. Although operation times are generally longer, some studies reported less morbidity and a shorter learning curve. Hinotori’s feasibility is evident; however, studies mostly report retrospective features, and there are still discussions about possible short-term advantages. Surgeons’ experiences in laparoscopic and robotic settings during these studies can impact the results. By experience, we mean both the handling of laparoscopic settings (unopen settings and similar robotic settings for the view and approach) and robot mechanisms. In most studies, retrospective laparoscopic data could have also contained historical data (from the less-experienced era of laparoscopic settings) or data from institutions with respect to learning curves for complicated cases [36]. On the other hand, the data for robotic procedures were mostly obtained within a short duration and from institutions that had good experiences in both laparoscopic settings and robotic handling. There should be robotic advantages for specific procedures, but this might not be the case for all procedures, especially if less-equipped robots (without CUSA, water jets, and other devices used in laparoscopic liver resection) and the bulky settings of surgical robots (those requiring wider areas between each port relative to the target) are considered. Our present report is important because this is the world’s first report on hinotori liver resection; moreover, the feasibility of carrying out robotic surgeries by a surgeon inexperienced in these types of surgeries was demonstrated (although backup from team staff with substantial experience in laparoscopic procedures was needed).

In the current settings, the main advantages of hinotori over da Vinci may be the larger working area around the port and lesser abdominal wall damage inflicted due to the docking-free design, in addition to the lower purchase price. In our present procedure, the Pringle maneuver, traction of the round ligament when moving the liver, and usage of staplers (in addition to occasional suction/irrigation/coagulation/sealing) were carried out by patient-side surgeons, in addition to the change in robot arm tools. Having a large area around the trocar is beneficial. Although we usually use CUSA/water jet devices in liver surgeries for liver transection in open and conventional laparoscopic settings, these are currently unavailable in robotic surgeries. Ultrasonic scalpels, used in our liver surgery, are also unbendable in robotic hands. A reduction in wound pain, which should result in “early recovery after surgery”, has been reported due to less damage inflicted on the abdominal wall from the “PIVOT” mechanism [10]. In Japan, the list price of da Vinci (Xi, single console) is JPY 276,000,000 (USD 1,820,000), while the double console version is priced at JPY 334,000,000 (USD 2,200,000). The annual maintenance costs are JPY 14,000,000 (USD 92,000) for the single console and JPY 17,500,000 (USD 115,000) for the double console. In comparison, hinotori is priced lower at JPY 235,000,000 (USD 1,550,000), with a maintenance fee of JPY 13,000,000 (USD 86,000) [12]. Hinotori also has a remote surgical function, and some reports of its experiments, including ours, have been published in recent years [37,38,39]. Hinotori was previously pointed out to have the following drawbacks: difficulties when confronted with floating sensations or motions, excessive emergency stop functions during arm collision, and a lack of hand-clutch functions [5,9,12]. The system has improved with updated software that reduces floating sensations and mitigates emergency stops, and hand-clutch functions have been added [5,40]. Recent meta-analyses using da Vinci showed that robot surgery resulted in comparable or better short-term outcomes compared to conventional laparoscopic liver resection [33,34,35]. The present study has several limitations, such as a small sample size, short-term follow-up periods, and an uncertain learning curve for the new robotic system. Future prospective investigations into the surgical outcomes of liver resection using hinotori are also needed.

## 5. Conclusions

This is the world’s first report of liver resection carried out using hinotori. The surgical settings and the cases were unique, which may have had an impact on the outcomes. However, hinotori liver resection was shown to be feasible. Several new platforms, including hinotori, are now entering this field. The advantages, disadvantages, indications, and learning curves of each system should be examined in future studies, which would result in future progression.

## Figures and Tables

**Table 1:** Characteristics of the 10 cases.

Case	Sex	Age	BMI	ASA	Disease	Operation	T Number	T Size (mm)	LC/LF ± PH	Surg Hx	Vessel Dissection
1	M	77	28.5	2	HCC	Partial resection (S4)	1	20	LC	1	1
2	F	60	31.2	2	HCC	Partial resection (S5-6)	1	14	LC + PH	0	1
3	M	69	24	2	hepatolithiasis	Lt hemi-hepatectomy + C	0	0	-	0	1
4	M	73	24	3	HCC	Partial resection (S4)	1	33	LC + PH	0	1
5	M	74	30.2	2	HCC	Partial resection (S5) + C	1	50	LF	0	1
6	F	72	15.2	3	met (pancreatic cancer)	Partial resection (S3-4)	1	10	-	1	0
7	M	67	22	3	met (rectal cancer)	Ext left medial sectionectomy + C	2	25	-	1	1
8	M	62	26.6	2	HCC	Partial resection (S7-8d)	2	10	LC	1	0
9	M	69	20.2	2	met (rectal cancer)	Partial resection (S2,2,6,6,8)	5	17	-	1	0
10	F	71	21.9	2	HCC	Partial resection (S7-8d) + C	2	11	LC + PH	0	0

M, male; F, female; BMI, body mass index; ASA, American Society of Anesthesiologists physical status; HCC, hepatocellular carcinoma; met, liver metastasis; +C, combined cholecystectomy; T, tumor; LC, liver cirrhosis; LF, liver fibrosis; +PH, with portal hypertension; Surg Hx, previous upper abdominal surgical history; vessel dissection, deep tumor with the need for major vessel dissection.

**Table 2:** Short-term outcomes of the 10 cases.

Case	Operation Time (Minutes)	Blood Loss (mL)	Conversion	LOS (Day)	Morbidity (II or Above)
1	260	26	0	5	0
2	327	80	0	7	0
3	577	330	0	7	0
4	433	212	0	7	0
5	402	1253	0	8	0
6	331	91	0	7	0
7	497	222	0	8	1
8	743	649	0	23	0
9	425	330	0	9	0
10	414	4081	1	9	0

LOS, postoperative length of hospital stays.

**Table 3:** Recent reports on short-term outcomes of robot-assisted liver resection [16,17,18,19,20,21,22,23,24,25,26,27,28,29,30,31].

Author (Year, Number of Cases)	Operation Time (Minutes)	Blood Loss (mL)	Conversion (%)	LOS (Day)	Morbidity (%)
Sijberden JP (2024, 1507)	190 (139–272)	100 (50–280)	3%	4 (3–6)	19% (III or above = 6%)
Krenzien F (2024, 461)	189 (140–271)	100 (50–200)	2%	5 (3–6)	20% (III or above = 6%)
Görgec B (2023, 400)	NA	150 (50–350)	6%	4 (2–5)	19% (III or above = 7%)
Huang XK (2024, 385)	NA	NA	NA	10 (3–59)	47% (III or above = 23%)
Winckelmans T (2023, 177)	145 (118–190)	30 (10–90)	1%	3 (1–3)	14% (III or above = 7%)
Di Benedetto F (2023, 158)	290 (190–380)	200 (100–500)	3%	4 (3–6)	54% (III or above = 6%)
Schmelzle M (2022, 129)	260 (83–568)	NA	5%	8 (4–94)	38% (III or above = 23%)
Sucandy I (2022, 125)	296 ± 117	253 ± 254	NA	5 ± 3	6% (II or above = 6%, III or above = 1%)
Li H (2024, 107)	212 (153–300)	100 (50–300)	3%	8 (7–9.5)	II or above = 4%
Fukumori D (2024, 100)	261 ± 113	194 ± 223	1%	4 ± 3	8% (III or above = 5%)
Steinkraus KC (2024, 100)	180 (IQR 128.7)	300 (IQR 550)	6%	6 (IQR4)	21% (III or above = 13%)
Chong CN (2020, 91)	259 ± 127	275 ± 568	8%	4.8 ± 1.8	10% (III or above = 3%)
Yang HY (2021, 70)	472 ± 203	270 ± 354	7%	10 ± 6	31% (II or above = 27%, III or above = 3%)
Kwak BJ (2023, 63)	233 (188–305)	100 (50–200)	2%	6 (5–8)	27% (III or above = 13%)
Chen W (2023, 48)	160 ± 62	92 ± 86	6%	5.4 ± 1.6	42% (II or above = 35%, III or above = 15%)
Birgin E (2024, 41)	223 (122–380)	300 (100–500)	5%	5 (4–7)	29% (III or above = 0%)

Robot-assisted procedure data are presented from 16 reports of robot-assisted liver resection between 2019 and 2024, retrieved from Pubmed, Embase, and Cochrane Library (excluding systematic reviews and meta-analyses). Data are expressed as median (range or IQR) or mean ± SD. LOS, postoperative length of hospital stays; NA, not available; II and III (in morbidity), Clavien–Dindo class II and III.

## Data Availability

Data are contained within the article and supplementary materials.

## Supplementary Materials

The following supporting information can be downloaded at https://www.mdpi.com/article/10.3390/jcm13247819/s1. Table S1: Patient’s demographic and clinical characteristics of 16 reports.
