# Supplementary material for: The First Report on Liver Resection Using the Novel Japanese hinotori™ Surgical Robot System: First Case Series Report of 10 Cases
Source: J Clin Med. 2024 Dec 21;13(24):7819. doi: 10.3390/jcm13247819 (PMC11727992; doi:10.3390/jcm13247819)
Supplement: Supplementary file 1 [file jcm-13-07819-s001.zip › jcm-3362736-supplementary.pdf]

Supplementary Table S1. Patient's demographic and clinical characteristics of 16 reports [16–31].

The data on robot-assisted procedures are from 16 reports of robot-assisted liver resection occurring between 2019 and 2024 and retrieved from Pubmed, Embase, and Cochrane Library (excluding systematic reviews and meta-analyses). Data are expressed as median (range or IQR) or mean +/- SD.

NA, not available; BMI, body mass index; ASA-PS, American Society of Anesthesiologists Physical Status

**Author (year, number of cases)**	**Sijberden J (2024,1507)**	**Krenzien F (2024,461)**	**Görgec B (2022,400)**	**Huang XK (2024,385)**	**Winckelmans T (2023,177)**	**Di Benedetto F (2022,158)**	**Schmelzle M (2022,129)**	**Sucandy I (2022,125)**	**Li H (2024,107)**	**Fukumori D (2024,100)**	**Steinkraus KC (2024,100)**	**Chong CN (2020,91)**	**Yang HY (2021,70)**	**Kwak BJ (2023,63)**	**Chen W (2023,48)**	**Birgin E (2024,41)**
**Sex (female)**	652(43%)	187 (41%)	179(45%)	67(17%)	69(39%)	33(21%)	63 (49%)	65(52%)	22 (21%)	48(48%)	41 (41%)	26 (29%)	26 (37%)	39(62%)	21 (44%)	21(51%)
**Age (years)**	62 (52-70)	61 (49-68)	64 (53-72)	NA	NA	66(58-71)	64 (22–85)	62(61±13.8)	53(11)	67(25–92)	61(IQR 14)	59±12	54±12	63 (53–69)	52±13	66 (56–75)
**BMI (kg/m2)**	26 (23- 29)	25 (23-28)	26(23-30)	NA	NA	26(24-29)	25 (17–40)	28(28±6)	25 (23–27)	26(17–39)	26(IQR 5)	25±4	24±3	NA	23±3	26 (23–27)
**ASA-PS≧3**	550 (37%)	173 (38%)	110(28%)	78(20%)	44(25%)	NA	64 (50%)	NA	30 (28%)	50(50%)	71(71%)	14 (15%)	NA	21 (33%)	2 (4%)	21 (51%)
**Pathology**																
Malignant	1100(73%)	379 (82%)	333(85%)	385(100%)	156(88%)	158(100%)	105(81%)	125(100%)	107(100%)	NA	65(65%)	NA	54(77%)	0	23 (48%)	41(100%)
Benign	407 (27%)	82 (18%)	62 (16%)	0	21(12%)	0	24 (19%)	0		22(22%)	11(11%)	NA	16(23%)	63(100%)	25 (52%)	0
**Extent of resection**																
Major	328 (22%)	NA	193(48%)	251(65%)	106(60%)	NA	61(47%)	125(100%)	NA	25(25%)	17(17%)	19 (21%)	70(100%)	32 (52%)	NA	10 (24%)
Minor	1179(78%)	NA	207(52%)	134(35%)	71(40%)	NA	68 (53%)	0	NA	75(75%)	83(83%)	72 (79%)	0	31(49%)	NA	29 (71%)
**IWATE difficulty score, median**	NA	NA	NA	NA	NA	6(4-8)	9 (1–12)	NA	NA	6±3	6 (IQR4)	NA	NA	NA	NA	8 (5–11)
Low	NA	0	NA	NA	NA	NA	NA	NA	16 (15%)	NA	23(24%)	NA	NA	0 (0%)	0	NA
Intermediate	NA	271 (59%)	NA	NA	NA	NA	NA	NA	59 (55%)	NA	41 (43%)	NA	NA	32 (51%)	12 (25%)	NA

≧Advanced)	NA	190 (41%)	NA	NA	NA	NA	NA	NA	30 (28%)	NA	32(33％）	NA	NA	31(49%)	36(75%)	NA
Advanced	NA	0	NA	NA	NA	NA	NA	NA	NA	NA	15 (16%)	NA	NA	28 (44%)	24 (50%)	NA
Expert	NA	190 (41%)	NA	NA	NA	NA	NA	NA	NA	NA	17 (18%)	NA	NA	3 (5%)	12 (25%)	NA
**Number of tumors**	NA	NA	1 (1-2)	ND	NA	NA	NA	NA	NA	NA	NA	NA	NA	NA	NA	NA
1	NA	NA	NA	NA	NA	NA	NA	NA	99 (93%)	NA	NA	NA	NA	NA	NA	NA
2	NA	NA	NA	NA	NA	NA	NA	NA	6 (6%)	NA	NA	NA	NA	NA	NA	NA
≧3	NA	NA	NA	NA	NA	NA	NA	NA	2 (2%)	NA	NA	NA	NA	NA	NA	NA
**Tumor size＞3 ㎝**	NA	NA	ND	NA	NA	NA	NA	NA	NA	NA	NA	NA	NA	NA	NA	NA
**Tumor size mm**	36 (22-60)	NA	27 (17-43)	NA	NA	NA	NA	50(50±37)	35(20–47)	40(0–200)	NA	NA	NA	NA	53±23	47 (24–80)
**Presence of cirrhosis**	377(25%)	141 (31%)	29 (7%)	282(73%)	NA	NA	17（13%）	NA	90 (84%)	NA	NA	NA	NA	2 (3%)	NA	4 (10%)
